# Integrative analysis of highly mutated genes in hepatitis B virus‐related hepatic carcinoma

**DOI:** 10.1002/cam4.2903

**Published:** 2020-02-04

**Authors:** Fanyun Kong, Delong Kong, Xiaoying Yang, Dongchen Yuan, Ning Zhang, Xuan Hua, Hongjuan You, Kuiyang Zheng, Renxian Tang

**Affiliations:** ^1^ Jiangsu Key Laboratory of Immunity and Metabolism Department of Pathogenic Biology and Immunology Xuzhou Medical University Xuzhou Jiangsu P. R. China; ^2^ National Demonstration Center for Experimental Basic Medical Sciences Education Xuzhou Medical University Xuzhou Jiangsu P. R. China

**Keywords:** gene mutation, hepatitis B virus, hepatocellular carcinoma, integrative analysis

## Abstract

Gene mutation is responsible for the development of hepatocellular carcinoma (HCC) with hepatitis B virus (HBV) infection; however, the characteristics and associated biological functions of highly mutated genes, in which the mutation frequencies are at least 5% in HCC patients with HBV infection, are not clearly evaluated. In the study, we analyzed the information regarding somatic mutation obtained by whole‐exome sequencing in 280 HBV‐related HCC tissues from public databases and published studies. Via integrative analysis, 78 genes, including TP53, TTN, MUC16, CTNNB1, and PCLO were summarized as highly mutated genes, and some of these mutated genes were further identified as cancer driver genes. Besides, we discovered that the highly mutated genes were enriched with various biological functions and pathways. The expression of many of highly mutated genes was found to be significantly altered in HBV‐related HCC, and several highly mutated genes were related to a variety of clinical factors and associated with the poor survival of the disease. Taken together, these results could enrich our understanding of highly mutated genes and their relationships with HBV‐related HCC. Some of the identified highly mutated genes might be used as novel biomarkers of disease prognosis, or as molecular targets for the treatment of HCC with HBV infection.

## Graphical abstract

The characteristics and associated biological functions of highly mutated genes, in which the mutation frequencies are at least 5% in HCC patients with HBV infection, are evaluated in the study, and these results could enrich our understanding of highly mutated genes and their relationships with HBV‐related HCC.

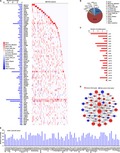

## 1 INTRODUCTION

Hepatocellular carcinoma (HCC), the major pathological type of liver cancer, is still a leading cause of cancer‐related death worldwide.^1^ In Asia and Africa, chronic hepatitis B virus (HBV) infection is the major cause of HCC development.^2^, ^3^, ^4^ Although it has been suggested that several factors,^5^, ^6^, ^7^ including the repeated inflammation mediated by immune reactions against the virus, the integration of HBV DNA into the host genome and the virus‐encoded oncoproteins (HBx and preS2/S proteins), are responsible for the development of HCC induced by HBV infection, the molecular mechanisms of hepatocarcinogenesis caused by HBV infection are still not well understanding, and no effective agents have been developed for the treatment of HBV‐related HCC. Hence, it is extremely important to discover novel molecules involved in the carcinogenesis of HBV‐associated HCC and offer the possibility of improving therapeutic approaches for HCC with HBV infection.

Accumulation of genetic alterations is a hallmark of HCC,^8^ and a great number of mutated genes, including CTNNB1, TSC1/2, PREX2, TP53, TERT, AXIN1, ARID1A, RET, and ARID2, have been identified in HCC, especially, in HBV‐related HCC.^3^, ^9^, ^10^, ^11^, ^12^, ^13^, ^14^, ^15^ Current studies suggest that the mutations of CTNNB1 are associated with low stage HCC.^16^ TERT promoter mutations are more frequently observed in HCC patients with low α‐fetoprotein (AFP) levels and advanced ages.^17^ Ho et al report that TSC1/2 mutations contribute to the disruption of the downstream mTOR activity and lead to the more aggressive behavior of HCC cells.^15^ In addition, special mutations in PREX2 gene are shown to have the capability of enhancing PREX2 protein stability and promoting cell proliferation.^18^ TP53 mutations are observed to be related to the chemotherapy resistance, tumor recurrence, and decreased overall survival (OS) of HCC patients.^19^ Besides these, the study from Ye et al shows that RET mutations are linked to poor disease free survival (DFS) and OS in patients with HCC.^20^ Taken together, these findings indicate that the mutations of special genes are related to specific pathological characteristics and abnormal biological functions of HCC, and play important roles in promoting HCC progression.

With the developing of sequencing technology, the mutations of several new genes, including APC, MYC, JAK1, COL11A1, LRP1B, FGF19, RB1, MUC16, and PCLO, in HCC tissues, especially in HBV‐related HCC tissues, have been discovered recently.^13^, ^21^, ^22^, ^23^ However, to date, a comprehensive understanding of the highly mutated genes, which are responsible for the development of HBV‐related HCC, is lacking. In the present study, based on the public databases and published studies, we integrated and analyzed the data of somatic mutations that obtained from whole‐exome sequencing in HBV‐related HCC, assessed the amount of highly mutated genes, investigated their biological functions and associated pathways, and further evaluated the relationship between these mutated genes with clinical‐pathological features and prognosis of HCC with HBV infection. Our findings will help us better understand the molecular mechanisms associated with gene mutations in the development of HBV‐related HCC.

## 2 MATERIALS AND METHODS

### 2.1 Data acquisition and processing

The somatic mutation information of HBV‐related HCC was obtained from cBioPortal database.^24^, ^25^ The somatic mutation information from different contributors was checked and analyzed if met the following conditions: (a) the detail somatic mutation data and clinical information of HBV‐related HCC could be available from the cBioPortal database or the published studies; (b) the same sequence method was used to detect the somatic mutations of enrolled samples. Finally, we integrated the somatic mutation data of 280 HBV‐related HCC from three different studies for further analysis,^22^, ^26^, ^27^ and the basic information of these three data is listed in Table 1.

**Table 1:** The characteristics of three studies selected from the cBioPortal database

Number of HBV‐related HCC	Sequencing platform	References
167	Whole‐exome sequencing	(AMC, Hepatology 2014)^22^
26	Whole‐exome sequencing	(INSERM, Nat Genet 2015)^26^
87	Whole‐exome sequencing	(TCGA, PanCancer Atlas)^27^

In the enrolled three studies, to obtain the somatic mutation information of HCC via whole‐exome sequencing, DNA was extracted from the frozen tissues of HCC, exome capture performed, paired‐end DNA sequence libraries generated, and then eluted‐enriched DNA sample was sequenced with the Illumina HiSeq 2000 platform.^22^, ^26^, ^27^ In each publication, the initial sequence analysis and the definition of mutation of DNA molecules were performed with MuTect, VarScan2, the GATK somatic indel detector, Mercury Pipeline, or Illumina pipeline.^22^, ^26^, ^27^ After the mutation data from these three publications were loaded into cBioPortal database,^24^, ^25^ the annotation of all mutation data provided by all studies was defined and standardized through a standard pipeline used in cBioPortal with Genome Nexus (https://genomenexus.org) and the canonical UniProt transcript (https://github.com/mskcc/vcf2maf/blob/master/data/isoform_ overrides_uniprot), via aligning reads to the human genome reference sequence hg19/GRCh37.

**Figure 1:**
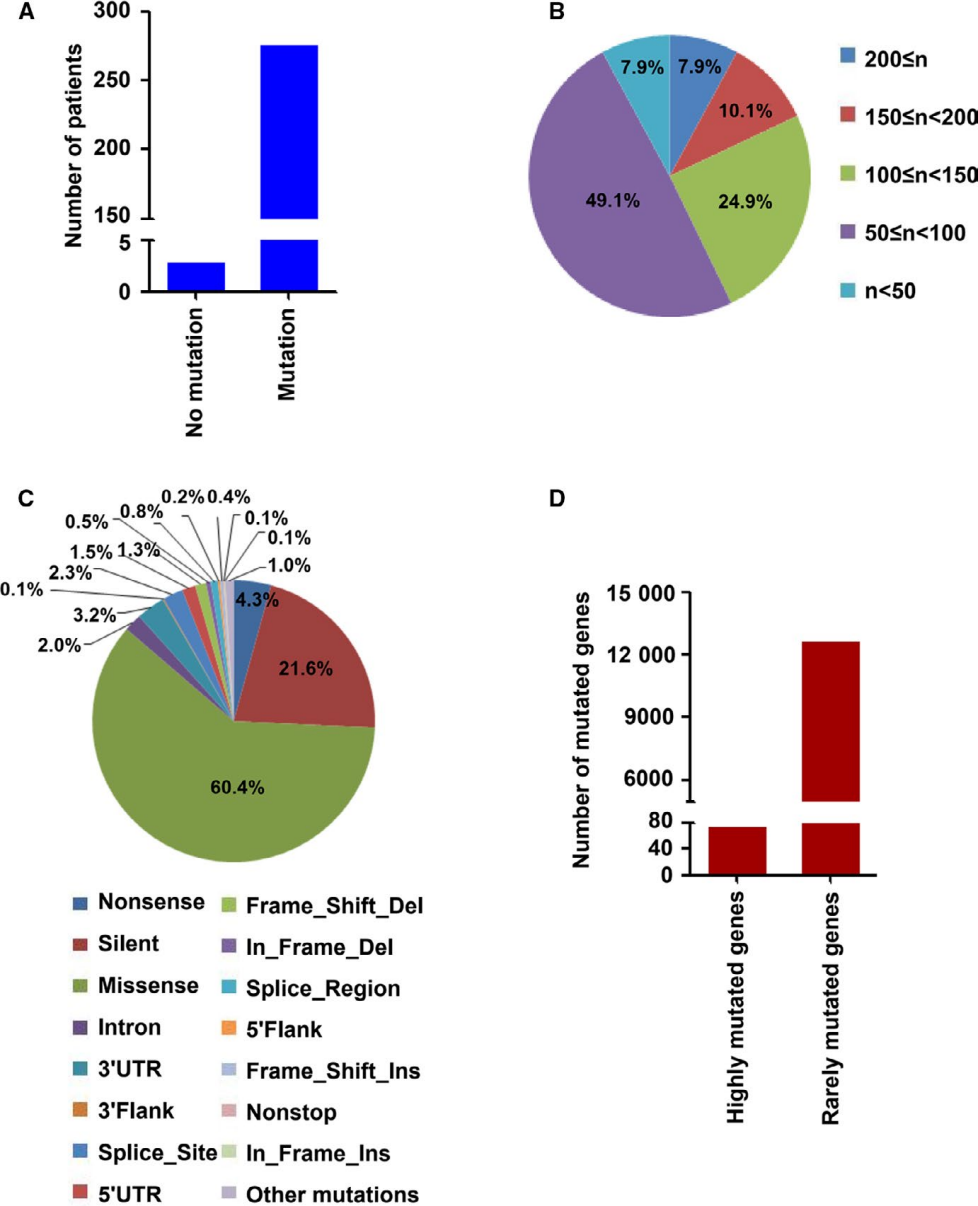
The mutation characteristics of recruited HBV‐related HCC. A, The number of HBV‐related HCC patients with or without somatic mutations. B, The number of gene mutations in HBV‐related HCC. C, The mutation types in HBV‐associated HCC. D, The number of identified highly mutated genes and rare mutated genes in HBV‐related HCC

After the standardized mutation data of HBV‐related HCC that obtained using the whole‐exome sequencing from these three studies were retrieved from cBioPortal database, the integrated analysis using bioinformatics methods was carried out in the present study. The preliminary processing of gene mutation data was performed with Excel 2007. Followed by the published studies,^28^, ^29^ the frequency of mutated target gene was no less than 5% in total samples was identified as highly mutated genes. Among these identified mutated genes in the study, 78 genes were considered as highly mutated genes, which had ≥5% mutation frequency (at least 14 patients had the mutation of target genes) in enrolled 280 HBV‐related HCC patients (Figure 1).

The gene expression profiling in FPKM (fragments per kilobase of exon per million reads) format that related to HBV‐related HCC tissues and non‐HCC tissues was retrieved from The Cancer Genome Atlas (TCGA) database (https://portal.gdc. cancer.gov/), analyzed with SPSS 19.0 software, and visualized with the heat map, which was drawn using the Heml software (Heatmap Illustrator, Version 1.0). This study was approved by the ethics committee of Xuzhou Medical University.

### 2.2 Gene function and pathway enrichment analysis

Gene ontology (GO) analysis was utilized to identify the molecular functions of enriched highly mutated genes.^30^ Kyoto Encyclopedia of Genes and Genomes (KEGG) pathway^31^ and WIKI pathway analysis,^32^ were carried out to determine the significant biological pathways with the clusters of highly mutated genes. In addition, GO analysis was measured with the g:Profiler online tool as described previously.^33^, ^34^ KEGG pathway and WIKI pathway analysis were performed using the ConsensusPathDB database.^34^, ^35^ For GO, KEGG, and WIKI enrichment analysis, the minimum enriched genes no less than 3, and a *P* value of < .05 was examined significant. In addition, the KEGG and WIKI pathway crosstalk analysis, based on the overlapped genes, was assessed via the online tool in ConsensusPathDB database,^35^ and visualized with the Cytoscape software.^36^ The overlap mutated genes no less than 3, *P* < .05 was considered as significant.

### 2.3 Protein‐protein interaction (PPI) network, cancer driver gene analysis, and data visualization

The PPI data of identified highly mutated genes were collected from STRING databases.^37^ Cancer driver gene analysis was performed with DriverDBv3, a database for human cancer driver gene research.^38^ Interaction networks of highly mutated genes were visualized using Cytoscape software.^36^ Bar graph and survival curve graph were made via Graphpad Prism software.^39^ Circular graph was presented using Excel 2007. Mutation data with different types in the enrolled HBV‐related HCC patients were produced using the open source program OncoPrinter (http://www.cbioportal. org/oncoprinter.jsp) in cBioPortal database,^24^, ^25^ and visualized with the heat map.

### 2.4 Validation of highly mutated genes based on the International Cancer Genome Consortium (ICGC) database

The ICGC database (https://icgc.org/) includes the information of somatic variants in “simple somatic mutation” format in cancer with different types. In order to validate the identified highly mutated genes, the somatic mutation information of LICA‐CN cohort in the ICGC database was used. In the LICA‐CN cohort, the somatic mutations in genomes in 402 patients with HBV‐associated HCC were available. In addition, the somatic mutation data of HBV‐related HCC were obtained by whole‐genome sequencing or random sequencing of exonic regions selected from the genome based on the Illumine HiSeq platform. However, the data associated with clinical information, including AFP, tumor size, vascular invasion, BCLC staging, and survival information cannot be obtained. The frequency of mutated target genes in total HBV‐related HCC patients was calculated and shown with the bar graph.

### 2.5 Statistical analysis

Statistical analysis was performed with SPSS 19.0 software (SPSS Inc). Survival analysis was performed with multivariate analysis with the Cox regression model, or Kaplan Meier analysis with log‐rank test. Data are presented as the mean ± SD or frequency in the study and analyzed using *t* test or chi‐square analysis. A value of *P* < .05 was regarded as significant.

## 3 RESULTS

### 3.1 The characteristics of gene mutations in HBV‐related HCC

To investigate the gene mutations in HBV‐related HCC, the whole‐exome sequencing data of 280 HCC patients with HBV infection, which were obtained from the cBioPortal database,^24^, ^25^ were integrated in this study. The clinical information of HBV‐related HCC patients is presented in Table 2 and Table S1, which was retrieved from cBioPortal databases and associated published studies. Among these enrolled HBV‐related HCC patients, only the samples from three patients were found to have no gene mutation (Figure 1A). We evaluated the number of mutated genes in HBV‐related HCC patients who had gene mutations. The results showed that most patients had mutations in multiple genes (Figure 1B and Table S2). For example, 49.1% patients had the number of mutated genes between 50 and 100. 24.9% patients had the number of mutated genes between 100 and 150. 10.1% patients had the number of mutated genes between 150 and 200 (Figure 1B). We also explored the mutation types of sequenced genes in HBV‐related HCC patients, and 16 types of gene mutations were found in these HBV‐associated HCC tissues. Missense mutation was the top 1 mutation type, which accounted for 60.4% of the total mutations. Silent type was the top 2 mutation type and constituted 21.6% of the total mutations. Other mutations were rare mutations that constituted less than 5% of total mutations (Figure 1C). Furthermore, among these mutated genes, 78 genes were recognized as highly mutated genes, the frequency of which accounted for no less than 5% of HBV‐related HCC patients. In addition, 12 726 genes were considered as rarely mutated genes, the percentage of which constituted less than 5% of HCC patients with HBV infection (Figure 1D).

**Table 2:** Baseline characteristics of HBV‐related HCC patients

Category	n/ mean ± SD
Gender (male/female)	211/69
Age (years)	53.2 ± 10.4
AFP (>20/≤20/NA, ng/mL)	158/119/3
Tumor size (>5/≤5/NA, cm)	48/145/87
Vascular invasion (Yes/No/NA)	98/179/3
BCLC staging (0‐A/B‐C/NA)	178/15/87

Abbreviation: NA, not available.

### 3.2 The information of highly mutated genes in HBV‐related HCC

**Figure 2:**
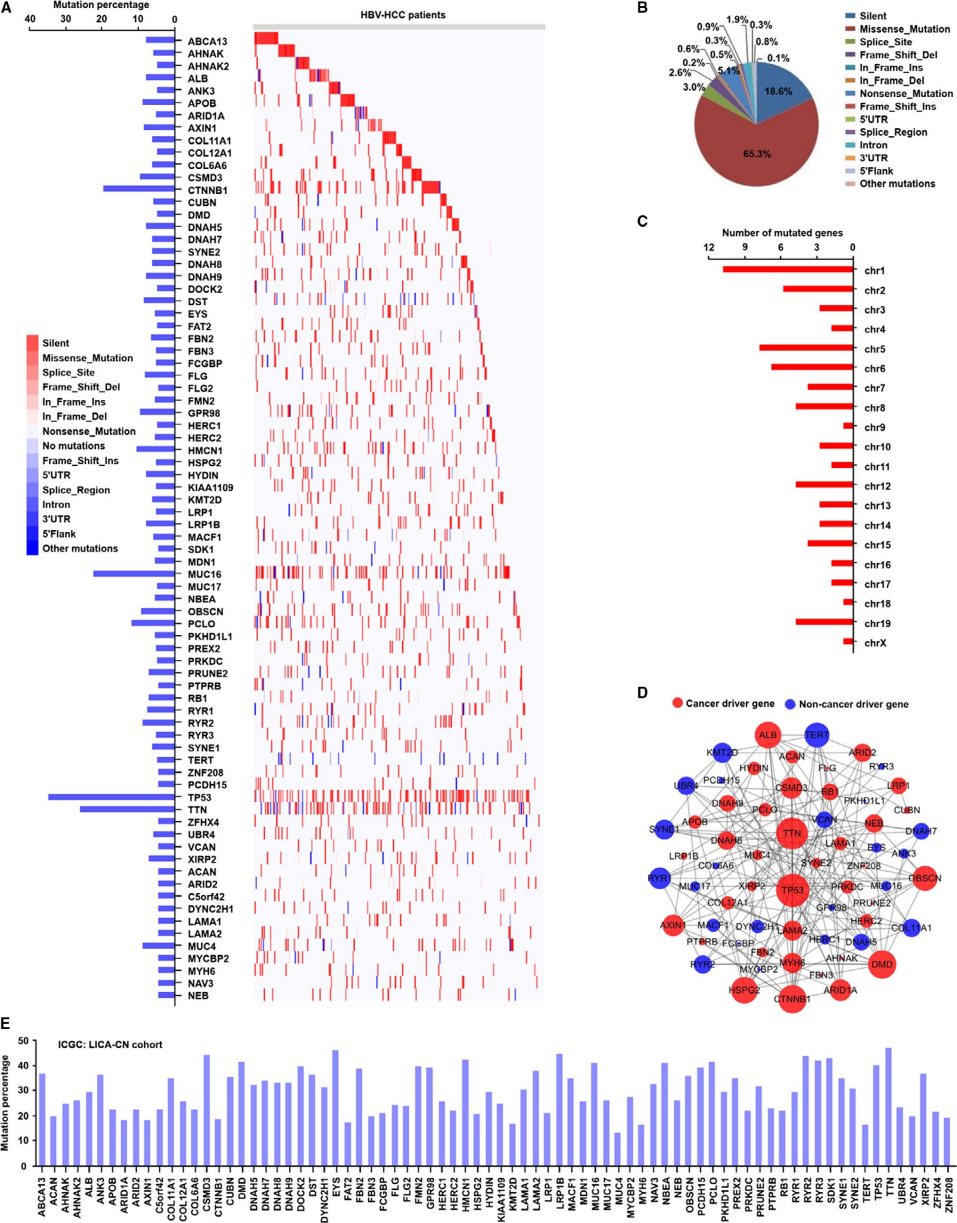
The information of highly mutated genes in HBV‐related HCC. A, The frequency and distribution of highly mutated genes in HBV‐related HCC. B, The mutation types of highly mutated genes in HBV‐related HCC. C, The chromosome distribution of highly mutated genes. D, The interaction of highly mutated genes, including identified cancer driver genes and noncancer driver genes, in the networks. E, The frequency of identified mutated genes in HBV‐related HCC in LICA‐CN cohort from ICGC database

The mutation frequency and mutation status of identified highly mutated genes in all enrolled HBV‐related HCC patients are shown in Figure 2A. Among these genes, TP53, TTN, MUC16, CTNNB1, PCLO, HMCN1, GPR98, CSMD3, OBSCN, and RYR2 were top 10 highly mutated genes in enrolled HBV‐related HCC patients. Meanwhile, 14 mutation types were found in these highly mutated genes, and the missense mutation, silent mutation, and nonsense mutation were the top 3 mutation types among the identified mutation types (Figure 2B). Other mutation types were rare mutations that accounted for less than 5% in total mutated genes (Figure 2B).

We next explored the chromosomal location of identified highly mutated genes in HBV‐related HCC patients. As the results are shown in Figure 2C, we observed that the highly mutated genes were widely distributed in 20 chromosomes (chr), from chr 1 to chr X. In addition, we found that a relatively high number of highly mutated genes were distributed in chr 1, chr 2, chr 5, chr 6, and chr 19.

In order to better understand the interaction of identified highly mutated genes, we constructed the interaction networks of these mutated genes, and PPI information was retrieved from STRING database.^37^ As shown in Figure 2D, the highly mutated genes constituted a large and complex network. Based on the degree of connection with other mutated genes, the top 10 hub highly mutated genes, including TP53, TTN, CNNTB1, ALB, TERT, DMD, OBSCN, CSMD3, AXIN1, and HSPG2, were found in the interaction network. To date, multiple cancer driver genes, which mutation could result in the development of different cancers, have been identified.^40^ In the study, we explored which mutated genes were cancer driver genes in HBV‐related HCC patients, using the DriverDBv3 online database.^38^ As the result shown in Figure 2D, 38 genes were identified as cancer driver genes and 24 genes as noncancer genes in PPI networks. In addition, TP53, TTN, DMD, CNNTB1, OBSCN, ALB, HSPG2, AXIN1, CSMD3, and ARID1A were identified as the top 10 cancer driver genes. TERT, SYNE1, KMT2D, DANH5, RYR1, RYR2, UBR4, DNAH7, COL11A1, and VCAN, were identified as the top 10 noncancer driver genes, in the interaction networks.

To further validate our identified mutated genes in HBV‐related HCC, the somatic mutation information of 402 HBV‐related HCC patients in LICA‐CN cohort from the ICGC database was used. As shown in Figure 2E, the mutation of identified 78 genes as mentioned above was found in HBV‐related HCC patients in LICA‐CN cohort. In addition, the mutation percentage in all identified 78 genes was no less than 5%, which was consistent with the results as presented in Figure 2A.

### 3.3 The molecular function and pathway enrichment analysis of highly mutated genes in HBV‐related HCC

**Figure 3:**
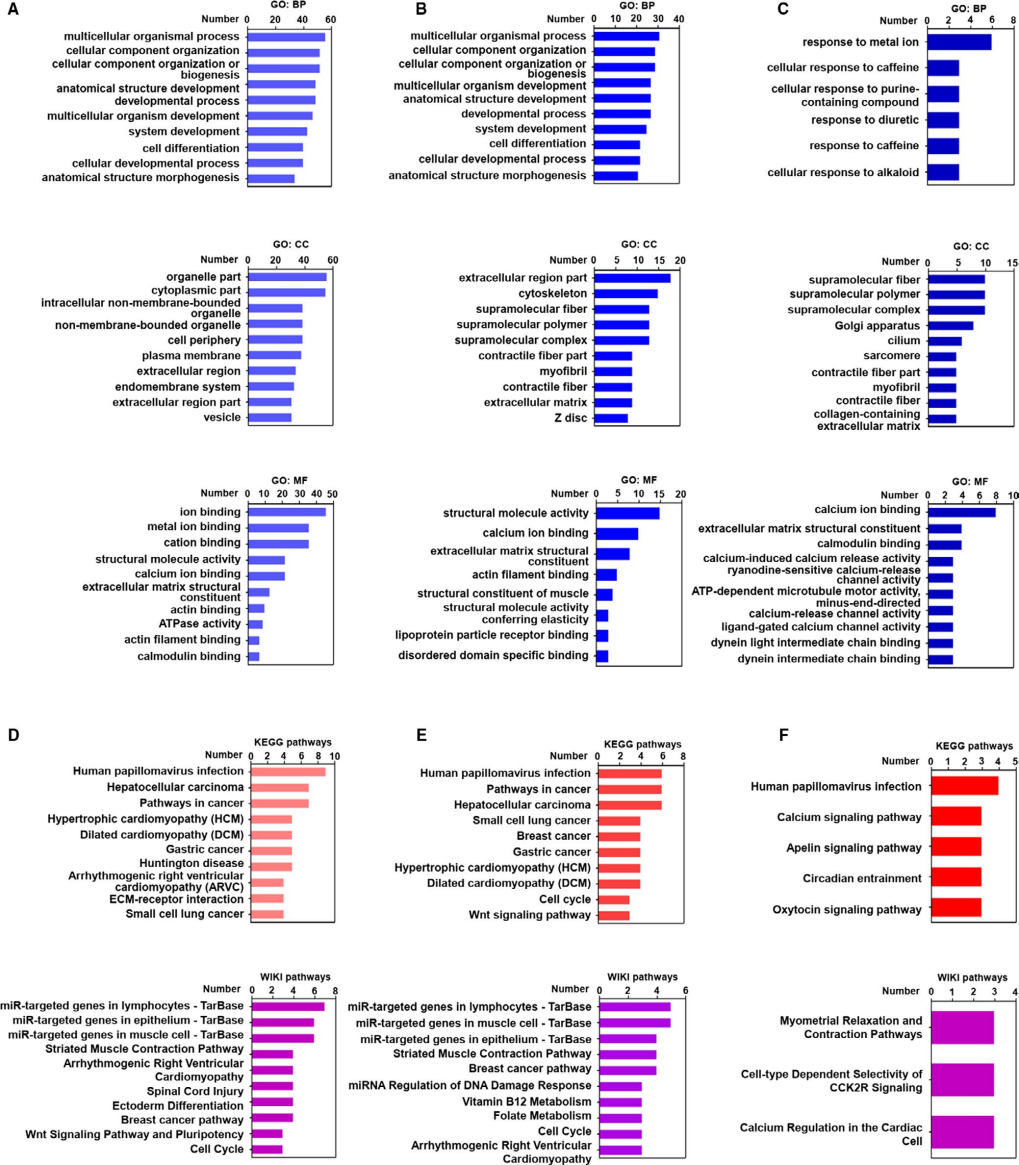
GO and pathway analysis of highly mutated genes, cancer driver genes and non‐cancer driver genes in HBV‐associated HCC. A, The top 10 GO terms of highly mutated genes in BP, CC, and MF. B, The top 10 GO terms of identified cancer driver genes in BP, CC, and MF. C, The top 10 GO terms of identified noncancer driver genes in BP, CC, and MF. D, The top 10 terms of KEGG pathways and WIKI pathways of highly mutated genes. E, The top 10 terms of KEGG pathways and WIKI pathways of cancer driver genes. F, The terms of KEGG pathways and WIKI pathways of noncancer driver genes

In order to investigate the molecular function of the highly mutated genes, GO analysis was performed using the g:Profiler online tool,^33^ and 112 significant GO terms were found (Table S3). According to the number of mutated genes, the top 10 enriched GO terms of the highly mutated genes were selected and presented in Figure 3A. The results of the GO analysis showed that the highly mutated genes were enriched in a variety of biological process (BP), cellular component (CC), and molecular function (MF). The enriched terms of BP in highly mutated genes were associated with multicellular organismal process, cellular component organization, cellular component organization or biogenesis, and anatomical structure development. The CC results showed that highly mutated genes were located in the organelle part, cytoplasmic part, cell periphery, and intracellular nonmembrane‐bounded organelle. In addition, the MF of enriched highly mutated genes was related to ion binding, structural molecule activity, extracellular matrix structural constituent, and actin binding.

We next explored the molecular function of identified cancer driver genes from highly mutated genes that shown in Figure 2D. A total of 69 significant GO terms were identified (Table S4). The top 10 enriched GO terms of the cancer driver genes are presented in Figure 3B. The results of the GO analysis show that the enriched terms of BP in cancer driver genes were associated with multicellular organismal process, cellular component organization, multicellular organism development, and anatomical structure development. The CC terms showed that cancer driver genes were located in the cytoskeleton, supramolecular fiber, supramolecular polymer, and supramolecular complex. The MF results showed that the enriched cancer driver genes were related to structural molecule activity, calcium ion binding, and extracellular matrix structural constituent.

The molecular function of identified noncancer driver genes in Figure 2D was also investigated by GO analysis (Table S5), and 39 significant GO terms were observed. We found that the enriched terms of BP in noncancer driver genes were associated with cellular response to caffeine, cellular response to purine‐containing compound, response to metal ion, diuretic, and alkaloid. The CC results showed that noncancer driver genes were situated in supramolecular fiber, supramolecular polymer, and supramolecular complex (Figure 3C). The enriched terms of CC in noncancer driver genes were associated with calcium ion binding, extracellular matrix structural constituent, and calmodulin binding.

To better investigate the biology pathways associated with the highly mutated genes, KEGG and WIKI pathway analysis were used based on the ConsensusPathDB database,^35^ and 28 significant KEGG pathway terms and 27 significant WIKI pathway terms were identified (Tables S6 and S7). According to the number of identified genes, the top 10 enriched KEGG pathways and WIKI pathways of highly mutated genes are shown in Figure 3D. The results of the KEGG pathways indicated that the highly mutated genes were associated with human papillomavirus infection, hepatocellular carcinoma, and the pathways in cancer. The terms of WIKI pathway suggested that the highly mutated genes were related to miR‐targeted genes in lymphocytes, epithelium, and muscle cell, striated muscle contraction pathways, and spinal cord injury.

In addition, 26 significant KEGG pathway terms and 22 significant WIKI pathway terms associated with cancer driver genes were found (Tables S8 and 9). The top 10 enriched terms of KEGG pathways and WIKI pathways related to cancer driver genes are displayed in Figure 3E. The results of the KEGG pathways indicated that the cancer driver genes were related to human papillomavirus infection, hepatocellular carcinoma, breast cancer, and gastric cancer. The terms of WIKI pathway showed that the cancer driver genes were associated with miR‐targeted genes in epithelium, lymphocytes and muscle cell, and breast cancer pathway.

A total of five significant KEGG pathway terms and three significant WIKI pathway terms related to noncancer driver genes were also noted (Figure 3F). The results of the KEGG pathways suggested that the noncancer driver genes were associated with human papillomavirus infection, circadian entrainment, apelin signaling pathway, oxytocin signaling pathway, and calcium signaling pathway. The terms of WIKI pathway showed that, the noncancer driver genes were relevant to myometrial relaxation and contraction pathways cell‐type dependent selectivity of CCK2R signaling, and calcium regulation in the cardiac cell.

### 3.4 The expression and associated molecular function analysis of highly mutated genes in HBV‐related HCC

**Figure 4:**
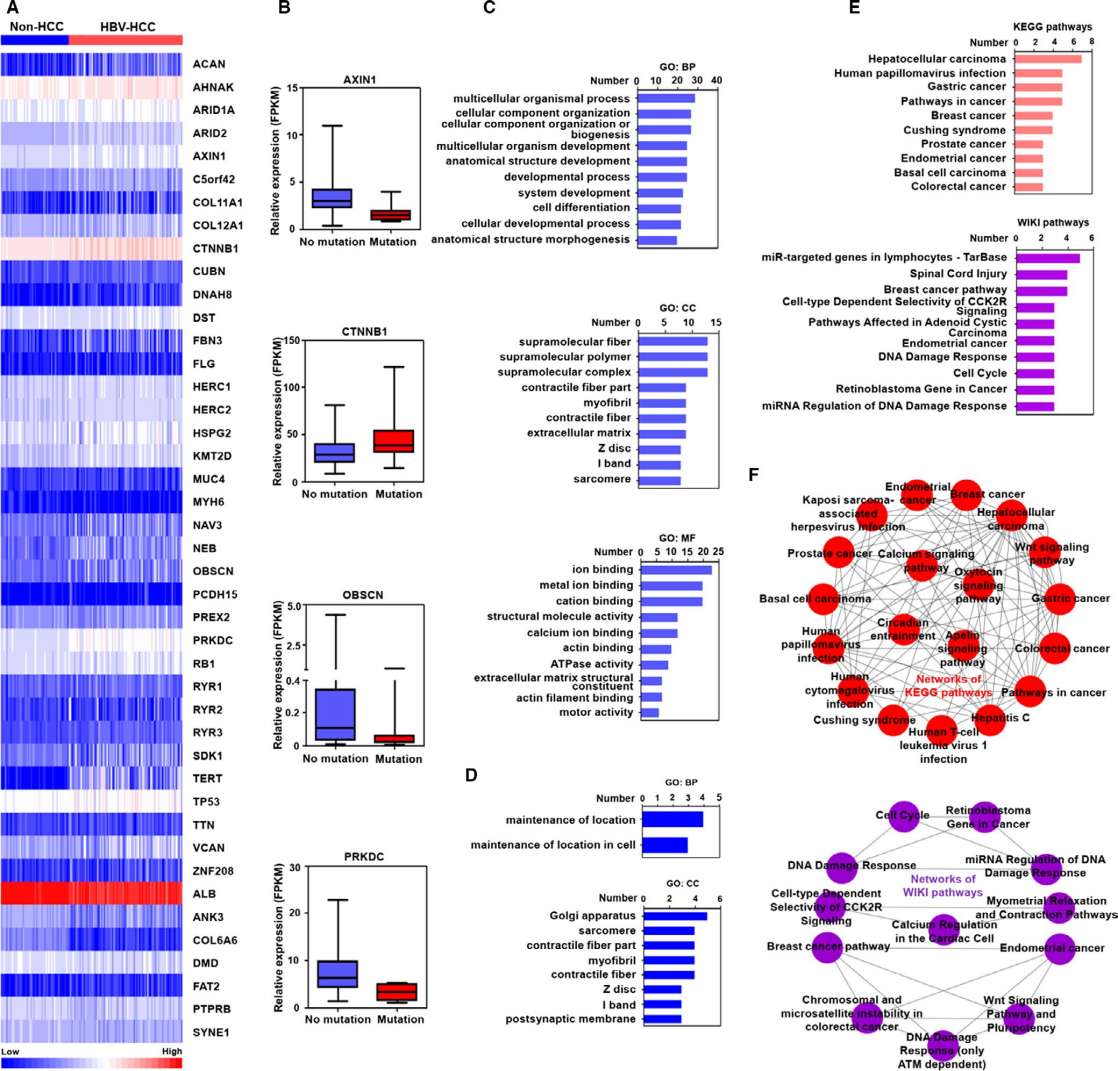
The gene expression and associated molecular function analysis of highly mutated genes in HBV‐related HCC. A, The significant expression of highly mutated genes in HBV‐related HCC from TCGA database. B, The effect of mutation on gene expression of highly mutated genes in HBV‐related HCC. C, The top 10 GO terms of upregulated mutated genes in BP, CC, and MF. D, The GO terms of downregulated mutated genes in BP and CC. E. The top 10 terms of KEGG pathways and WIKI pathways of upregulated mutated genes. F, The crosstalk of KEGG pathways and WIKI pathways associated with upregulated mutated genes

We evaluated the expression profile of highly mutated genes in HBV‐related HCC tissues that retrieved from TCGA database. As shown in Figure 4A, compared with non‐HCC tissues, the expression levels of 43 highly mutated genes were significantly altered in HBV‐HCC tissues. Among these genes, the expression levels of 35 genes, including AHNAK, ARID1A, AXIN1, COL11A1, COL12A1, and CNNTB1 were increased in the HBV‐associated HCC tissues. The expression levels of eight genes, including ALB, ANK3, COL6A6, DMD, FAT2, MYH6, PTPRB, and SYNE1 were downregulated in HBV‐related HCC.

We also investigated whether the mutation has an effect on the expression of identified genes in HCC tissues. The results showed that most of the somatic mutations of identified genes had no significant effect on the expression of themselves. However, the mutation of ATXN1, OBSCN, and PRKDC could cause the decreased expression of themselves. The mutation of CTNNB1 could increase self‐expression (Figure 4B).

We also explored the molecular function of upregulated mutated genes and downregulated mutated genes via GO analysis. A total of 96 significant GO terms of upregulated mutated genes were found (Table S10), and the top 10 enriched GO terms of upregulated mutated genes are displayed in Figure 4C. The results of the GO analysis indicated that the enriched terms of BP in upregulated mutated genes were associated with multicellular organismal process, cellular component organization, and cell differentiation. The CC results showed that upregulated mutated genes were located in supramolecular fiber, supramolecular polymer, and myofibril. The MF of enriched upregulated mutated genes was related to ion binding, metal ion binding, and cation binding.

The molecular function of downregulated mutated genes was also investigated by GO analysis. The enriched terms of BP in downregulated mutated genes were associated with maintenance of location, and maintenance of location in cell. The enriched terms of CC in downregulated mutated genes were related to sarcomere, contractile fiber part, myofibril, contractile fiber, and postsynaptic membrane (Figure 4D).

Next, a total of 22 significant KEGG pathway terms and 16 significant WIKI pathway terms were identified in upregulated mutated genes (Table S11 and 12). The top 10 enriched KEGG and WIKI pathways of upregulated mutated genes are presented in Figure 4E. The results of the KEGG pathways indicated that the upregulated mutated genes were associated with hepatocellular carcinoma, gastric cancer, and human papillomavirus infection. The terms of WIKI pathway showed that the upregulated mutated genes were related to miR‐targeted genes in lymphocytes, breast cancer pathways, Wnt pathway and pluripotency, and cell cycle. No significant enriched KEGG and WIKI pathways were identified in downregulated mutated genes.

Different pathways are capable of influencing each other via a phenomenon called crosstalk. Especially, it is evident that the pathways could affect each other, when there is an overlap of differentially expressed genes, which have significant changes at expression level.^41^, ^42^ Therefore, identification of the interaction of different pathways based on the overlap of differentially expressed genes has important implications for the understanding of the molecular mechanisms associated with the development of HBV‐related HCC. In the present study, according to the overlap of upregulated mutated genes between different pathways, the complex crosstalk networks of KEGG pathways and WIKI pathways associated with upregulated mutated genes were structured (Figure 4F).

### 3.5 The association of highly mutated genes with the clinical factors in HBV‐related HCC

**Figure 5:**
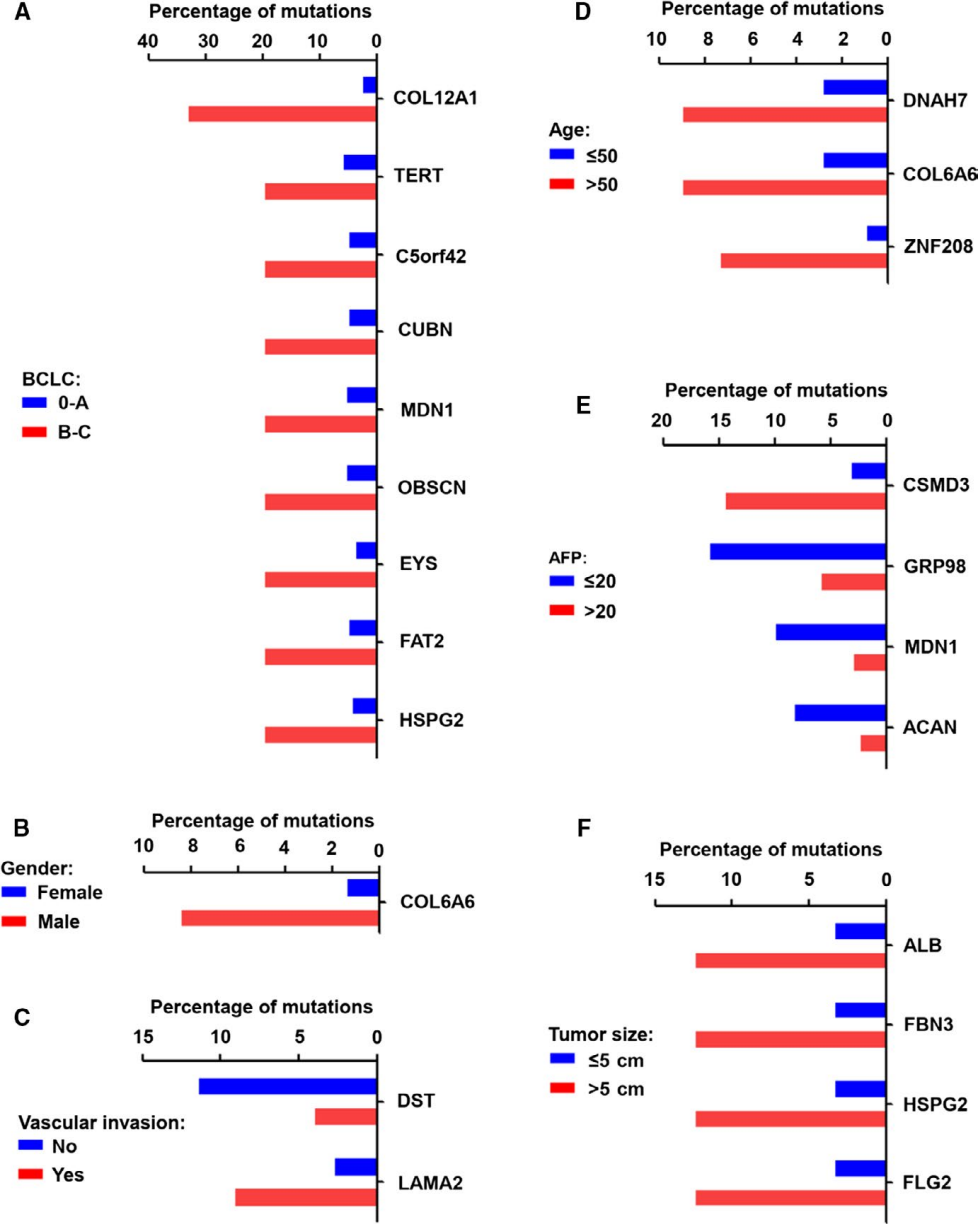
The relationship of highly mutated genes with various clinical factors in HBV‐related HCC patients. A, The significant frequency of highly mutated genes with different BCLC stages in HBV‐related HCC patients. B, The significant frequency of highly mutated genes in male and female HBV‐related HCC patients. C, The significant frequency of highly mutated genes with or without vascular invasion in HBV‐related HCC patients. D, The significant frequency of highly mutated genes with different ages in HBV‐related HCC patients. E, The significant frequency of highly mutated genes with different AFP levels in HBV‐related HCC patients. F, The significant frequency of highly mutated genes with different tumor sizes in HBV‐related HCC patients

We further assessed the association of highly mutated genes with different clinical parameters, including BCLC stage, gender, AFP, age, vascular invasion, and tumor size, in HBV‐related HCC. As the results shown in Figure 5A, compared with HBV‐related HCC with BCLC staging between 0 and A, the mutation frequencies of COL12A1, TERT, c5orf42, CUBN, MDN1, OBSCN, EYS, FAT2, and HSPG2 were increased in patients with BCLC staging between B and C. In addition, compared with female HBV‐related HCC patients, the mutation percentage of COL6A6 was significantly increased in male HCC patients with HBV infection (Figure 5B). In HBV‐associated HCC patients with vascular invasion, the mutation frequency of DST was decreased, the mutation frequency of LAMA2 was increased, compared to patients without vascular invasion (Figure 5C). In HBV‐related HCC patients with age more than 50, the mutated frequencies of DNAH7, COL6A6, and ZNF208 were increased, compared to patients with age no more than 50 (Figure 5D). Compared to patients with AFP levels no more than 20, CSMD3 mutation percentage was significantly increased, but the mutation frequencies of GPR98, MDN1, and ACAN were decreased in patients with AFP levels more than 20 (Figure 5E). Compared with patients with tumor size no more than 5 cm, the mutation percentages of ALB, FBN3, HSPG2, and FLG2 were increased in patients with tumor size more than 5 cm (Figure 5F).

### 3.6 The relationship of highly mutated genes with the DFS and OS of HBV‐related HCC patients

**Figure 6:**
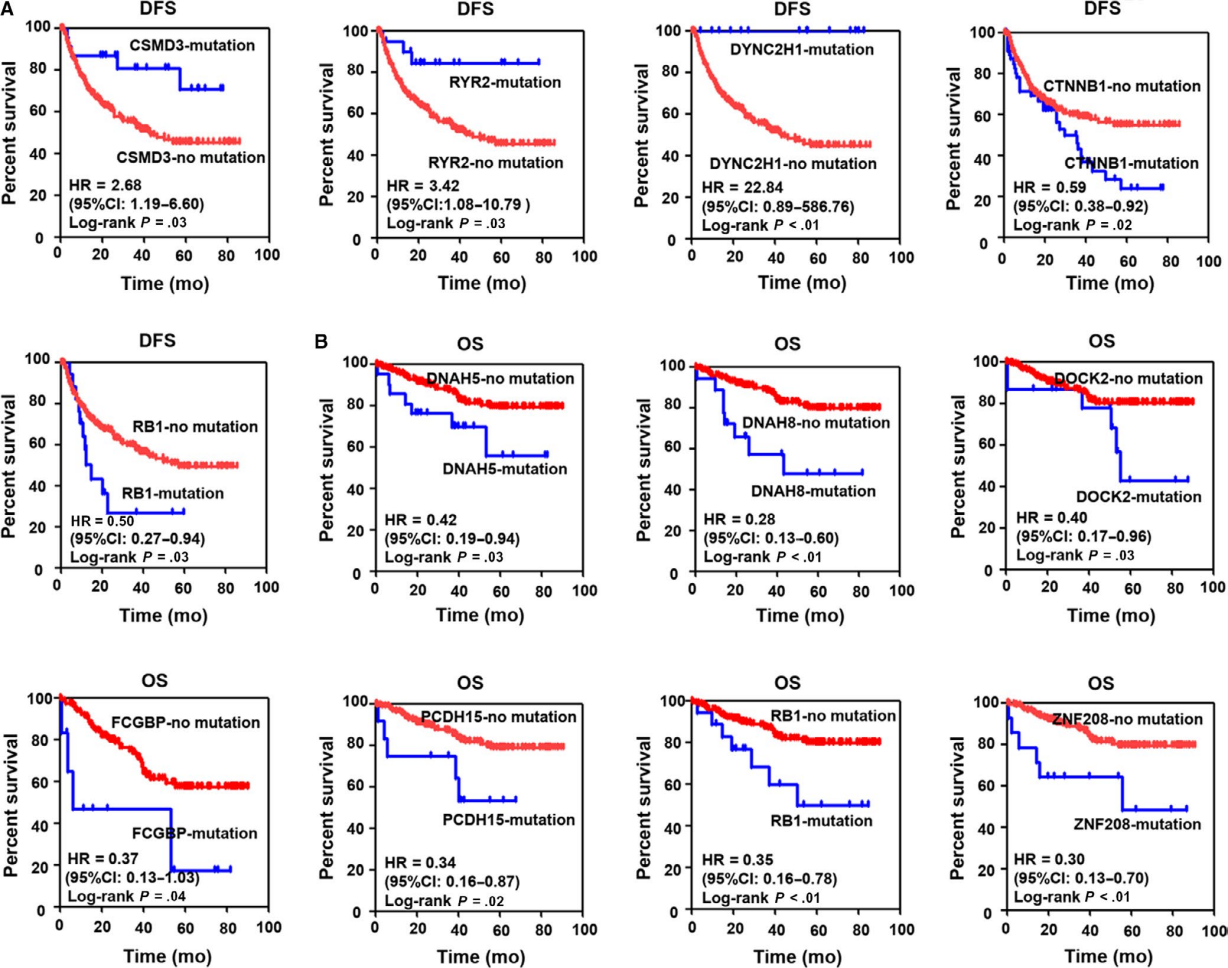
The association of highly mutated genes with the survival in HBV‐related HCC based on the Kaplan‐Meier analysis with log‐rank test. A, The relationship of CSMD3, RYR2, DYNC2H1, CTNNB1, and RB1 mutations with DFS in HBV‐related HCC. B, The relationship of DNAH5, DHAH8, DOCK2, FCGBP, PCDH15, RB1, and ZNF208 mutations with OS in HBV‐related HCC. HR: Hazard radio; 95CI: 95% confidence interval

Using the Cox regression model in the multivariate analysis, we explored the relationship of highly mutated genes with two clinical outcomes, including DFS and OS, in patients with HBV‐related HCC. The results showed that, among these highly mutated genes, the mutations of many genes, including CTNNB1, DMD, RB1, and RYR2 were related to the poor DFS of HBV‐related HCC (Table 3). In addition, several genes, including AHNAK, ANK3, COL12A1, DNAH8, HERC2, NBEA, and RB1 were related to poor OS of HBV‐associated HCC (Table 4). We also assessed the correlation of the highly mutated genes with the survival of HBV‐related HCC patients using Kaplan‐Meier analysis with log‐rank test. As shown in Figure 6A. The CSMD3, RYR2, and DYNC2H1 mutations were associated with beneficial DFS of HBV‐related HCC patients. CTNNB1 and RB1 mutations were associated with the poor DFS of HCC patients with HBV infection. In addition, the mutations of DNAH5, DNAH8, DOCK2, FCGBP, PCDH15, RB1, and ZNF208, were related to the poor OS of HBV‐related HCC patients (Figure 6 B).

**Table 3:** The significant association of highly mutated genes with the DFS of HBV‐related HCC patients based on Cox regression analysis

Covariates	Hazard radio	95% confidence interval	*P* value
ACAN	0.037	0.006‐0.246	.001
ALB	3.185	1.128‐8.990	.029
ANK3	0.193	0.041‐0.909	.038
APOB	3.584	1.295‐9.922	.014
ARID1A	3.106	1.125‐8.577	.029
CTNNB1	3.432	1.516‐7.766	.003
DMD	5.659	1.500‐21.352	.011
NDAH8	3.081	1.050‐9.042	.041
FBN3	0.187	0.036‐0.975	.047
FLG	3.088	1.037‐9.197	.043
HSPG2	0.145	0.022‐0.969	.046
LRP1B	3.909	1.420‐10.756	.008
MUC17	4.448	1.099‐17.999	.036
MYCBP2	0.107	0.018‐0.634	.014
RB1	4.239	1.687‐10.655	.002
RYR2	0.038	0.008‐0.175	.001
TTN	1.919	1.020‐3.610	.043

**Table 4:** The significant association of highly mutated genes with the OS of HBV‐related HCC patients based on Cox regression analysis

Covariates	Hazard radio	95% confidence interval	*P* value
AHNAK	33.073	2.926‐373.812	.005
ANK3	0	0‐0.073	.009
APOB	30.148	2.524‐360.088	.007
ARID1A	8.206	1.050‐64.152	.045
c5orf42	4062.075	50.948‐323868.972	.001
COL11A1	165.465	4.907‐5579.938	.004
COL12A1	12.950	1.961‐85.506	.008
COL6A6	27.758	1.173‐656.916	.040
CSMD3	0.007	0‐0.305	.010
CUBN	0.059	0.003‐0.979	.048
DNAH5	58.966	3.260‐1066.640	.006
DNAH8	122.162	10.764‐1386.407	.001
HECR2	0.001	0‐0.357	.022
LRP1	0.027	0.001‐0.638	.025
NBEA	0.019	0.001‐0.539	.020
PCDH15	427.813	17.808‐10277.640	.001
RB1	13.032	1.728‐98.309	.013
ZNF208	29.594	3.064‐285.830	.003

**Figure 7:**
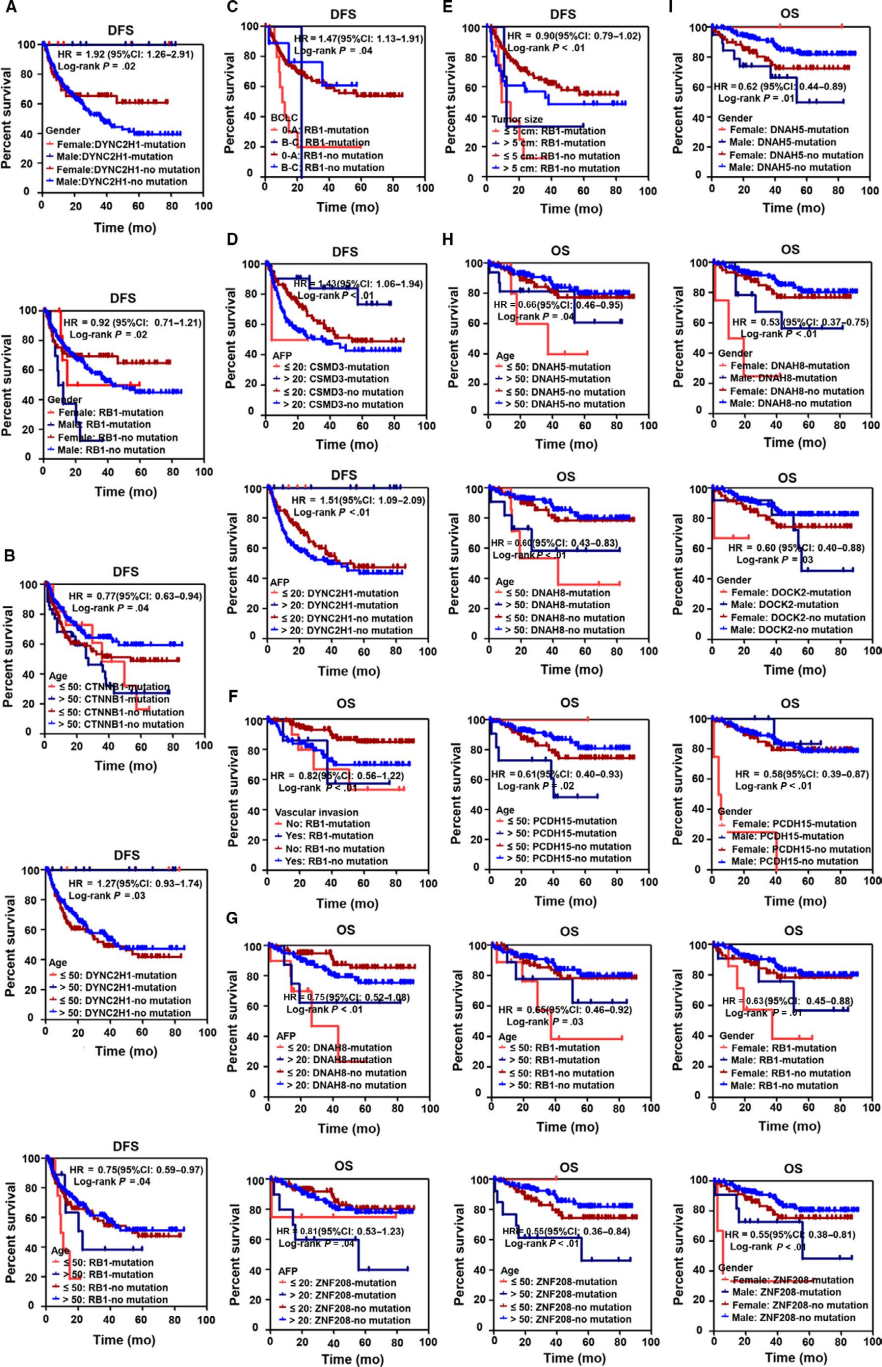
The association of highly mutated genes and different clinical parameters with survival in HBV‐related HCC patients based on the Kaplan‐Meier analysis. A, The association of DYNC2H1, RB1 mutation, and gender with DFS in HBV‐related HCC. B, The association of CTNNB1, DYNC2H1, RB1 mutation, and age with DFS in HBV‐related HCC. C, The association of RB1 mutation and BCLC staging with DFS in HBV‐related HCC. D, The association of CSMD3, DYNC2H1 mutation, and AFP with DFS in HBV‐related HCC. E, The association of RB1 mutation and tumor size with DFS in HBV‐related HCC. F, The association of RB1 mutation and vascular invasion with OS in HBV‐related HCC. G, The association of DNAH8, ZNF208 mutation, and AFP with OS in HBV‐related HCC. H, The association of DNAH5, DNAH8, PCDH15, RB1, ZNF208 mutation, and age with OS in HBV‐related HCC. I, The association of DNAH5, DNAH8, DOCK2, PCDH15, RB1, ZNF208 mutation, and gender with OS in HBV‐related HCC. HR: Hazard radio; 95CI: 95% confidence interval

Based on different clinical parameters, we also explored the subgroup analyses to detect the association of the highly mutated genes and clinical parameters with OS or DFS in HBV‐related HCC patients. The results showed that the DYNC2H1 mutation with female or male was associated with beneficial DFS of HBV‐related HCC patients. RB1 mutation with female or male was associated with poor DFS of HBV‐related HCC patients (Figure 7A). CTNNB1 mutation with age ≤ 50 or > 50 was associated with poor DFS of HBV‐related HCC patients. DYNC2H1 mutation with age ≤ 50 or > 50 was associated with beneficial DFS of HBV‐related HCC patients. RB1 mutation with age ≤ 50 or age > 50 was associated with poor DFS of HBV‐related HCC patients (Figure 7B). RB1 mutation with BCLC 0‐A or BCLC B‐C was associated with poor DFS of HBV‐related HCC patients (Figure 7C). CSMD3 mutation with AFP ≤ 20 or CSMD3 no mutation with AFP > 20 was associated with poor DFS of HBV‐related HCC patients. DYNC2H1 mutation with AFP ≤ 20 or > 20 was associated with beneficial DFS of HBV‐related HCC patients (Figure 7D). RB1 mutations with tumor size ≤ 5 cm or tumor size > 5 cm were associated with poor DFS of HBV‐related HCC patients (Figure 7E).

In addition, RB1 mutations with vascular invasion or without vascular invasion were associated with poor OS of HBV‐related HCC patients (Figure 7F). DNAH8 mutation with AFP ≤ 20 or AFP > 20 was associated with poor OS of HBV‐related HCC patients. ZNF208 mutation with AFP ≤ 20 or > 20 was associated with poor OS of HBV‐related HCC patients. (Figure 7G). DNAH5 mutation with age ≤ 50 or age > 50 was associated with poor OS of HBV‐related HCC patients. DNAH8 mutation with age ≤ 50 or age > 50 was associated with poor OS of HBV‐related HCC patients. PCDH15 mutation with age > 50 or PCDH15 no mutation with age ≤ 50 was associated with poor OS of HBV‐related HCC patients. RB1 mutation with age ≤ 50 or age > 50 was associated with poor OS of HBV‐related HCC patients. ZNF208 mutation with age > 50 or ZNF208 no mutation with age ≤ 50 was associated with poor OS of HBV‐related HCC patients (Figure 7H). DNAH5 mutation with male or DNAH5 no mutation with female was associated with poor OS of HBV‐related HCC patients. DNAH8 mutation with female or male was associated with poor OS of HBV‐related HCC patients. DOCK2 mutation with female or male was associated with poor OS of HBV‐related HCC patients. PCDH15 mutation with female or PCDH15 no mutation with male was associated with poor OS of HBV‐related HCC patients. RB1 mutation with female or male was associated with poor OS of HBV‐related HCC patients. ZNF208 mutation with female or male was associated with poor OS of HBV‐related HCC patients (Figure 7I).

## 4 DISCUSSION

Genetic aberration contributes to the progression of HCC with HBV infection^8^; however, the relationship of highly mutated genes with the development of HBV‐related HCC is not fully clarified. In the present study, we obtained the information regarding somatic mutations in HBV‐related HCC from the public databases and published studies. Based on the integrated analysis, the highly mutated genes with multiple mutation types were found, and many highly mutated genes were further identified as cancer driver genes. In addition, these highly mutated genes exerted distinct molecular functions and were associated with a variety of pathways. Furthermore, the expression levels of multiple highly mutated genes were altered in HBV‐related HCC tissues, and some highly mutated genes were related to different clinical factors and associated with the poor survival of HBV‐related HCC.

HBV infection could cause genomic instability, including mutation and epigenetic misregulation in genes,^43^ and multiple mechanisms are involved in gene mutation mediated by the virus. For example, HBV is capable of interfering with the function of mitotic regulatory proteins and mitotic spindle checkpoint proteins, which have the role of maintaining genomic integrity.^3^, ^44^ The virus could integrate the viral genome into the host DNA to induce genetic alterations.^44^ Persistent infection of the virus creates an inflammation environment that could induce the aggregation of genetic mutations.^45^ In this study, based on the integration and analysis of the mutation data of HBV‐related HCC produced by whole‐exome sequencing, we found that HBV could cause a great number of gene mutations with multiple types in most of the HCC patients. In addition, a lot of genes only had rare mutations, whose frequency was less than 5% of total patients. We identified 78 highly mutated genes that associated with HBV‐related HCC, and the results were further validated by using the LICA‐CN cohort in ICGC database. Among these identified highly mutated genes, TP53, TERT, PEEX2, CTNNB1, and AXIN1 in HCC, especially in HBV‐related HCC, have been investigated, and the published studies showed that these mutated genes played very important roles in the development of HCC.^10^, ^11^, ^18^, ^21^ In addition, consistent with the published studies, we found that COL11A1, RB1, MUC16, and PCLO, which have been detected by next‐generation sequencing technology,^13^, ^21^, ^22^, ^23^ including whole genome sequencing and whole‐exome sequencing by different groups, are also found to be associated with HCC in our research. However, the role of other mutated genes, including ABCA13, COL11A1, and COL12A1 as shown in Figure 2A, in HBV‐related HCC, has not been well clarified and needs to be investigated in future studies. Furthermore, the molecular characteristics and associated mechanisms of these identified highly mutated genes have been not well assessed. In order to define the genetic abnormalities that are related to HBV‐related HCC, we mainly focused on the identified highly mutated genes and explored their potential biological function and associated pathways in HBV‐related HCC via bioinformatics approaches.

The somatic mutation could cause changes in gene function, and the mutation with distinct types has different roles in target genes.^46^ We explored the mutation types in highly mutated genes and found that the predominant mutation types are missense mutation, silent mutation, and nonsense mutation. Missense mutation is a mutation with a single‐nucleotide substitution in a gene. This mutation could result in an amino acid substitution in the encoded protein and further lead to the change of biological function in the target gene.^47^ Silent mutation is a kind of mutation, which is outside of genes or in regulatory elements of a gene. Silent mutation cannot change the coding region of target genes, but it has a very important effect on gene regulation from transcription, mRNA stability to translation or splicing.^48^ Nonsense mutation is a single‐nucleotide substitution in the target gene, and this mutation would lead to the production of a stop codon.^47^ Although different mutation types were identified, the functional significance of these mutations in the identified genes on HBV‐related HCC cells is still unclear. Additional studies are required to investigate the role and associated molecular mechanisms of these mutations in HBV‐related HCC.

Chromosomal aberrations have been demonstrated in HCC, especially in HBV‐related HCC.^10^ Deletion and amplification of distinct genes in special chromosomes were identified by copy number variation (CNV) analysis in HCC.^49^, ^50^ For example, the gains of CNVs in chr 1, chr 6, chr 11, and chr 20, and losses in chr 8, chr 9, and chr 13.^50^, ^51^, ^52^ In order to determine whether the distribution of the highly mutated genes was associated with special chromosomes, we detected the location of these highly mutated genes in the genomes of HCC. We found that a relatively high number of highly mutated genes were located on five chromosomes, including chr 1, chr 2, chr 5, chr 6, and chr 19. These results indicate that the development of HCC with HBV infection is related to the genetic alteration, including CNVs and somatic mutations, in specific chromosomes.

We next investigated the interaction of identified mutated genes, and large and complex interaction networks were constructed. These results suggest that the highly mutated genes could promote HBV‐related HCC progression via the interaction with each other. In addition, several hub highly mutated genes with a high degree of connectivity were identified in the interaction networks. Furthermore, in interaction networks, many mutated genes were identified as cancer driver genes. The cancer driver gene is identified as a gene that contains driver mutations, which could cause a selective growth advantage to the cell, and lead to tumorigenesis.^53^ It is also shown that most cancers carry more than one driver gene and the number of driver genes varies among different cancer types.^40^ Until now, a variety of driver genes have been identified using various computational methods that mainly dependent on the mutation frequency of an individual gene in cancers.^54^ Base on the DriverDBv3,^38^ a cancer driver gene identification database, many highly mutated genes, including TP53, TTN, DMD, ALB, and OBSCN, were identified as cancer driver genes in HCC in the study. These results suggest that the mutations of identified genes were positively selected and facilitated the growth of cells during the evolution of HBV‐related HCC. In addition, many noncancer driver genes, such as TERT, KMT2D, and RYR1, are also found in Figure 2D. Furthermore, we found that many of the identified cancer driver genes and noncancer driver genes could interact with others. These results indicate that, in a coordinated manner, these identified cancer driver genes and noncancer driver genes might play very important roles in the development of HCC with HBV infection.

In order to better explore the biological functions related to HBV‐related HCC, GO analysis was performed, and the results show that the identified mutated genes were associated with various BP, CC, and MF. The BP of highly mutated genes was associated with multicellular organismal process, cellular component organization, and anatomical structure development. In addition, the highly mutated genes were found to locate in various cellular areas, including cytoplasmic part, organelle part, and cell periphery. These results indicate that the proteins encoded by these mutated genes in different cellular areas might have distinct biological roles. In addition, the MF of the highly mutated genes was related to ion and cation binding, and structural molecule activity. The molecular function of identified cancer driver genes from highly mutated genes was also analyzed. The results show that the enriched GO terms of BP in cancer driver genes were related to cellular component organization, multicellular organism development, and anatomical structure development. The CC results showed that cancer driver genes were located in cytoskeleton, supramolecular fiber, supramolecular polymer, and supramolecular complex. The MF of enriched cancer driver genes was found to be related to structural molecule activity, calcium ion binding, and extracellular matrix structural constituent. Besides these, the molecular function of noncancer driver genes that shown in Figure 2D was also identified via GO analysis. The results showed that the enriched GO terms of BP in noncancer driver genes were related to cellular response to caffeine, cellular response to purine‐containing compound, response to diuretic, caffeine, and alkaloid. The CC of GO terms indicated that noncancer driver genes were located in supramolecular fiber, supramolecular polymer, and supramolecular complex. The MF of enriched noncancer driver genes was found to be related with calcium ion binding, and calmodulin binding. These GO terms enriched by identified mutated genes, including cancer driver genes and noncancer driver genes, could allow us to further explore the biological functions of highly mutated genes in HBV‐related HCC.

The development of HBV‐related HCC is considered to be implicated with several distinct molecular pathways.^45^ Based on KEGG pathways, we discovered that significant pathways were related to different cancers, indicating that these identified mutated genes might play critical roles in the development of multiple types of cancers. The mutated genes associated with Wnt signaling pathways and cell cycle pathways have been reported to facilitate the development of HCC.^3^, ^45^ Consistent with published studies, based on WIKI pathways, we found that the pathways related to Wnt signaling and cell cycle were enriched by the identified mutated genes in HBV‐related HCC. In addition, the significant pathways related to identified cancer driver genes were investigated. The results of the KEGG pathways indicated that cancer driver genes were related to hepatocellular carcinoma, breast cancer, and gastric cancer. The terms of WIKI pathway showed that the cancer driver genes were associated with miR‐targeted genes in epithelium, lymphocytes and muscle cell, and breast cancer pathways. Besides these, the results of the KEGG pathways indicated that noncancer driver genes were associated with circadian entrainment, apelin signaling pathway, and oxytocin signaling pathway. The terms of WIKI pathway showed that the noncancer driver genes were related to cell‐type dependent selectivity of CCK2R signaling, calcium regulation in the cardiac cell and myometrial relaxation and contraction pathways. The development of HBV‐related HCC is a complex multistep process, and our results suggested that several distinct molecular pathways related to highly mutated genes, including cancer driver genes and noncancer driver genes, may be implicated.

Recently, the aberrant expression of distinct genes has been reported in HBV‐related HCC tissues, and we were interested in investigating the expression of the highly mutated genes in HBV‐related HCC tissues. By analyzing the gene expression profiles of HBV‐related HCC and non‐HCC tissues from TACG database, the expression of a total of 43 highly mutated genes was found to be altered. Among these genes, AHNAK, ARID1A, AXIN1, RB1, and P53 have been reported to inhibit the proliferation and invasion of HCC cells.^55^, ^56^, ^57^, ^58^ However, the expression of these genes was increased in HBV‐related HCC as shown in Figure 4A. These results implied that increased expression of these mutated genes might cause the loss of function on tumor suppression, and contribute to the development of HCC. Other identified genes, including CTNNB1, PREX2, and TERT,^59^, ^60^, ^61^ facilitate the survival or invasion of HCC cells. In the study, we found that these three genes were upregulated in HCC. Furthermore, current studies indicated that the mutation of these genes could promote their expression, stability, or activity to facilitate the development of HCC. For example, the mutation of TERT in the promoter region could increase the expression of TERT gene.^62^ PREX2 gene mutation could enhance the stability of PREX2 protein.^18^ In addition, specific CTNNB1 mutations could enhance the activity of ß‐catenin that associated with malignant transformation in HCC.^63^ In addition, our results show that the mutation in most of the identified genes, but not AXIN1, CTNNB1, OBSCN, and PRKDC, had no significant role on the expression of target genes, implying that the most of mutations on genes mainly have an effect on gene function but not their expression in HBV‐related HCC.

We also explored the molecular function of identified upregulated mutated genes and downregulated mutated genes via GO, KEGG pathways and WIKI pathways. The results suggested that the enriched GO terms of BP in upregulated mutated genes were associated with the multicellular organismal process and cellular component organization. The CC terms indicated that upregulated mutated genes were located in supramolecular fiber and supramolecular polymer. The MF terms of enriched upregulated mutated genes were shown to be related to ion binding, metal ion binding, and cation binding. In addition, the enriched GO terms of BP in downregulated mutated genes were associated with maintenance of location. The enriched GO terms of CC in downregulated mutated genes were related to sarcomere, contractile fiber part, and myofibril. These distinct enriched GO terms associated with upregulated mutated genes and downregulated mutated genes implied that these identified dysregulated mutated genes maybe have a vital role in the progression of HBV‐related HCC.

In addition, KEGG pathway analysis indicated that the upregulated mutated genes were associated with hepatocellular carcinoma, gastric cancer, and human papillomavirus infection. WIKI pathway showed that, the upregulated mutated genes were related to miR‐targeted genes in lymphocytes, breast cancer pathways, and endometrial cancer. Given that upregulated mutated genes and downregulated mutated genes were associated with a variety of pathways, targeting these specific pathways identified in the study might be a potential strategy for HBV‐related HCC. Furthermore, previous studies mainly focused on the role of a single pathway associated with mutated genes on HBV‐related HCC.^45^ The contribution of the interactions among distinct pathways that related to the mutated genes on the development of HBV‐associated HCC is not properly assessed. Via pathway crosstalk analysis based on overlapping upregulated mutated genes in different identified pathways, we found that the KEGG pathways and WIKI pathways could form complex interaction networks based on the overlap of upregulated mutated genes. These results suggest that the identified pathways with upregulated mutated genes may play important roles in HBV‐related HCC in a coordinated manner. Further understanding of the significant dysfunction crosstalk between identified pathways of upregulated mutated genes will help us to provide intense insights into the molecular mechanisms of HCC with HBV infection.

The association of highly mutated genes with clinical factors and survival of HBV‐related HCC was further assessed in the study. The results indicate that many mutated genes were associated with distinct clinical factors, including BCLC stage, gender, age, AFP, vascular invasion, and tumor size. Furthermore, the results of multivariate analysis with the Cox regression model show that many mutated genes, including CTNNB1, DMD, PRKDC, RYR2, and RB1 were associated with DFS of HBV‐related HCC. AHNAK, ANK3, COL12A1, and RB1 were related to the OS of HBV‐related HCC. In addition, based on the Kaplan‐Meier analysis with log‐rank test, the mutations of CTNNB1 and RB1 were found to be related to the poor DFS of HBV‐related HCC. DNAH5, DHAH8, and DOCK2 mutations were associated with the OS of HBV‐related HCC. Furthermore, many gene mutations associated with different clinical parameters are also related to beneficial or poor DFS or OS of HBV‐related HCC. These results suggest that the mutated genes, or combined with mutated genes with different clinical parameters, could be used as biomarkers of the clinical prognosis of HCC patients with HBV infection. Moreover, integration of the mutation information of these identified genes with suitable methods may be a useful strategy for monitoring and management of HBV‐related HCC to reduce the mortality of HCC patients with HBV infection.

In summary, via integrative analysis of the mutation data from HBV‐related HCC, we identified 78 highly mutated genes that were involved in the development of HCC with HBV infection. Our results suggest that these highly mutated genes have multiple mutation types, could form complex networks, and some of them are further identified as cancer driver genes. In addition, these mutated genes are associated with distinct biological functions and pathways. Furthermore, various mutated genes are significantly associated with clinical factors and related to the poor progress of HBV‐related HCC. Given the functional and clinical significance of highly mutated genes, these mutated genes could serve as novel therapeutic targets or molecular biomarkers for HBV‐related HCC patients. Besides these, our study has a few limitations. For instance, the molecular functions and associated pathways of identified highly mutated genes were only generated from bioinformatics analysis. The functional importance of different mutations in the identified genes needs to be further elucidated in HBV‐related HCC, based on in vitro and in vivo experiments in future studies. Despite the limitations, our present study still lays a foundation for further exploring the roles and associated molecular mechanisms mediated by the identified mutated genes in the HCC with HBV infection.

## CONFLICT OF INTEREST

The authors declare that they have no conflict of interest.

## Data Availability

The data that support the findings of this study are available from the corresponding author upon reasonable request.
